# Follicle-Stimulating Hormone Glycosylation Variants Distinctly Modulate Pre-antral Follicle Growth and Survival

**DOI:** 10.1210/endocr/bqac161

**Published:** 2022-10-06

**Authors:** Gillian P Johnson, Caitlan G A Onabanjo, Kate Hardy, Viktor Y Butnev, George R Bousfield, Kim C Jonas

**Affiliations:** Department of Women and Children's Health, School of Life Course and Population Health Sciences, Kings College London, London SE1 1UL, UK; Institute of Reproductive and Developmental Biology, Imperial College London, London W12 0NN, UK; Department of Women and Children's Health, School of Life Course and Population Health Sciences, Kings College London, London SE1 1UL, UK; Institute of Reproductive and Developmental Biology, Imperial College London, London W12 0NN, UK; Department of Biological Sciences, Wichita State University, Wichita, KS 67260, USA; Department of Biological Sciences, Wichita State University, Wichita, KS 67260, USA; Department of Women and Children's Health, School of Life Course and Population Health Sciences, Kings College London, London SE1 1UL, UK

**Keywords:** follicle stimulating hormone, follicle stimulating hormone receptor, follicle, ovary, menopause, aging

## Abstract

Follicle-stimulating hormone (FSH) is a key endocrine regulator of ovarian function. FSH is secreted as 2 macroglycosylation variants: partially glycosylated FSH (FSH21/18) and fully glycosylated FSH (FSH24). FSH21/18 is more potent than FSH24 at binding to and activating the FSH receptor (R). The ratio of FSH21/18:FSH24 has been shown to change with age, with FSH21/18 predominant at reproductive prime, and FSH24 predominant during perimenopause/menopause. How these FSH glycosylation variants modulate ovarian follicle functions remains largely unknown. The aim of this study was to investigate the effect of FSH glycosylation variants of pre-antral follicle function. Pre-antral follicles were isolated from 3- to 5-week-old C57BL/6 mice and treated ±10 ng/mL FSH21/18, FSH24, a ratio of 80:20 FSH21/18:FSH24 (to mimic reproductive prime), 50:50 FSH21/18:FSH24 (perimenopause), or 20:80 FSH21/18:FSH24 (menopause) for up to 96 hours. FSH21/18 and 80:20 FSH21/18:FSH24 increased follicle growth, in comparison with control, contrasting with FSH24 and 20:80 FSH21/18:FSH24. Survival rates were decreased in follicles treated with FSH24 or 20:80 FSH21/18:FSH24, with follicles undergoing basement membrane rupture and oocyte extrusion, increased Caspase3 gene and protein expression, and decreased markers of cell proliferation in FSH24 or 20:80 FSH21/18:FSH24–treated follicles. Moreover, this correlated with differential regulation of key genes modulating follicular functions. Pharmacological inhibitors of key FSH signal pathways suggests FSH21/18 and FSH24 initiate different FSHR signal pathway activation, which may determine their differential effects on follicle growth and survival. These data suggest that the nature of FSH glycosylation modulates the follicular cellular environment to regulate follicle growth and survival and may underpin the increasing ovarian resistance to FSH observed during aging.

Female reproductive aging and eventual ovarian senescence occurs decades prior to the decline of any other human organ system (1–3). One of the hallmarks of ovarian aging is the increase in circulating levels of follicle-stimulating hormone (FSH) because of decreased ovarian negative feedback from inhibin B as the ovarian reserve depletes (4). Clinically, this decline in ovarian reserve is associated with a truncated follicular phase and shortened menstrual cycle (5), and ultimately reduced fertility/infertility. The mid-life cessation of ovarian function increases susceptibility to several comorbidities, including diabetes, cardiovascular disease, osteoporosis, impaired cognitive function, and metabolic syndrome (6, 7). Yet, little is known about the role of endocrinological mechanisms that contribute to the aging process.

FSH and its G protein–coupled receptor (FSHR) are essential for reproductive function. A plethora of evidence from knockout mouse studies (8, 9) and human mutations in either FSH or FSHR (10–12) has shown their actions to be essential for the transition of pre-antral to antral follicles, and for antral follicle development in the mammalian ovary, where FSHR expression is initiated in the granulosa cells (GCs) of growing pre-antral follicles (13–15). Functionally, FSH stimulates the proliferation of GCs via the FSHR (16–19) and estradiol secretion (20), resulting in increased follicle growth as a result of GC proliferation, and not increased oocyte size (18, 21, 22), supporting follicle maturation.

FSH and FSHR signal via canonical and noncanonical pathways. While it is widely agreed that the cellular and physiological actions of FSH are mediated by the activation of the G*α*s/adenylyl cyclase (AC)/cyclic adenosine monophosphate (cAMP)/protein kinase A (PKA) pathway, such as the induction of aromatase expression and control of ovarian steroidogenesis (23–25), it is becoming increasingly apparent that FSH/FSHR can activate multiple signal pathways in vitro and in vivo (25, 26). Indeed, previous research in primary and immortalized GCs have suggested gonadotropin receptor–mediated mechanisms that regulate signal cross-talk to direct steroidogenesis and programmed cell death (27, 28). Moreover, FSH has been shown to activate phosphatidylinositol-3-kinase/protein kinase B signaling in GCs to modulate follicle atresia (29), with additional signal pathway activation and cross-talk observed (25, 26, 30), supporting the signal and functional complexity of FSH/FSHR in directing follicle growth and development.

In addition to pleiotropic signaling identified by FSH/FSHR, glycosylation variants of FSH have also been identified, the production of which are modulated by age and menstrual cycle. FSH is a heterodimeric glycoprotein hormone comprising 2 noncovalently associated subunits: an α-subunit common to other glycoprotein hormone family members (thyrotropin, luteinizing hormone, human chorionic gonadotropin) and a hormone-specific β-subunit, which confers biological activity and specificity to FSHR (31). Previous studies have identified different FSH glycoforms in postmortem human pituitary extracts and/or urine, with 3 major FSH glycoforms identified based on the presence of 1 or 2 N-glycans in the FSHβ subunit (32–36). Glycosylation of both Asn7 and Asn24 FSHb residues was designated fully glycosylated FSH (FSH24, based on detection of a 24-kDa FSHβ band on immunoblots), with 2 additional glycoforms variants classified as hypoglycosylated or partially glycosylated FSH, FSH21/18 based on the presence of a single FSHb glycan at either Asn7 or Asn24, resulting in 21- and 18-kDa immunoreactive bands, respectively (34). These FSH glycoforms have been reported to be modulated by age, with an age-dependent decrease in the abundance of FSH21/18 (37, 38). Indeed, FSH21/18 predominates in younger women with ovulatory cycles, with FSH24 increasing with age and is predominant in perimenopausal/menopausal women (33–35, 38, 39). Functionally, FSH glycoforms display differences in binding to FSHR (34, 35) and in the timing, duration, and magnitude of signal pathways activated, with FSH21/18 more efficacious and potent than FSH24 (40–43). The age-dependent change in FSH glycoform abundance is significant in the context of ovarian aging not only due to the overall rise in levels of FSH at perimenopause, but also due to the differences in bioactivity between these glycoforms.

While FSH glycoforms have been shown to change with aging, their remains a knowledge gap in understanding their role(s) in modulating folliculogenesis. The objective of this study, therefore, was to determine how FSH21/18 and FSH24 regulate the growth and survival of cultured murine preantral follicles, when follicles become FSH sensitive (22). To mimic the aging-dependent changes in FSH21/18 and FSH24 ratios, follicles were cultured in FSH21/18, a ratio of FSH21/18:FSH24 at 80:20 (reproductive prime), 50:50 (perimenopause), and 20:80 (menopausal), and FSH24. Predominance of FSH21/18 promoted pre-antral follicle growth survival and steroidogenic gene expression, in contrast to predominance of FSH24, which was detrimental for follicle growth and survival, with distinct signal pathways implicated in mediating these functional differences. Together, these suggest that aging-dependent modulation of FSH glycoforms may adversely affect pre-antral follicle growth and survival. This has wider implications in the context of declining ovarian function observed during perimenopause.

## Materials and Methods

### Tissue Collection, Follicle Isolation, and Culture

Whole ovaries were collected from C57BL/6 female mice aged 3-5 weeks (Harlan, Shardlow, UK). Mice were housed in accordance with the Animals (Scientific Procedures) Act of 1986 and associated Codes of Practice. Ovaries were removed, and ∼50 follicles/ovary were mechanically isolated using acupuncture needles and placed in Leibovitz L15 medium (11415064; Life Technologies, Paisley, UK) supplemented with 1% (weight/volume) bovine serum albumin (A2153, Sigma) and 1% penicillin/streptomycin sulfate (P0781, PS; Sigma). Individual follicles were then transferred into a single well (1 follicle per well) in a 96-well plate containing 100 mL minimal essential medium alpha (11590606; Life Technologies) supplemented with 0.1% bovine serum albumin, 1% PS, and a cocktail of 5 mg/mL insulin, 5 mg/mL transferrin, and 5 ng/mL sodium selenite (41400-045, Sigma) as previously described (22). Isolated follicles from each ovary were distributed randomly and evenly between treatments in a single 96-well plate, with 6 to 8 follicles were cultured per treatment group for each plate. Follicle viability at plating was estimated >95%. Follicles were incubated in a humidified incubator in 5% CO_2_ at 37 °C for up to 96 hours. Follicles were photographed daily, and follicle area or diameter was measured using ImageJ 1.45 seconds (https://imagej.nih.gov/ij/) at each time point.

### Recombinant FSH Preparations and Treatment

Individual recombinant human FSH glycoforms—fully-glycosylated FSH24 and hypo-glycosylated FSH21/18—were purified from rat pituitary GH_3_ cell culture media co-expressing fully glycosylated FSH24 and hypoglycosylated FSH21/18. The purification and characterization of the glycoforms from GH_3_-conditioned media was described in detail elsewhere (35, 39). In brief, serum-free GH3-conditioned medium was collected, centrifuged, and loaded on anti-alpha subunit immunoaffinity column 4882. The column was washed with equilibration buffer (25 mM Sodium Phosphate, pH 7.5) until the baseline was stable, and hFSH was eluted with 0.1 M Glycine-HCl, 0.5 M NaCl, pH 2.7, neutralized, and concentrated on UltraCel-15 (Amicon). The separation of the glycoforms was achieved by gel filtration on a Superdex-75 triple column, fractions collected, dried, and analyzed by Coomassie gel and Western blotting using anti-equine beta subunit rabbit polyclonal antibodies. Based on the Western blot results, the fractions at the beginning of the heterodimer peak and at the end of the peak were pooled as hFSH24 (fully glycosylated) and hFSH21/18 (hypoglycosylated) preparations, respectively. Follicles were treated ±10 ng/mL FSH21/18, FSH24, a ratio of FSH21/18:FSH24 at 80:20 (to mimic reproductive prime), 50:50 (perimenopause), or 20:80 (menopausal).

### Signaling Pathway Inhibitor Culture

Follicles were pretreated with either 90 μL of medium alone containing vehicle (0.1% (v/v) DMSO), 1 μM H89 (PKA inhibitor), 100 nM Wortmannin (PI3K inhibitor), or 1 μM U0126 (MAPK inhibitor) for 30 minutes. Following pretreatment, 10 μL of medium alone (control), 100 ng/mL FSH21/18 or FSH24, was added to the corresponding 90 μL of the pretreated well, to give a final concentration of 10 ng/mL FSH glycoforms. Follicles were subsequently cultured for up to 96 hours. Follicles were snap frozen for RNA isolation.

### RNA Isolation, cDNA Synthesis and Real-time qPCR Assays

At the end of each appropriate culture period, approximately 5 follicles per treatment group were pooled into 1 tube and snap frozen in liquid nitrogen. Follicles were lysed with TRI Reagent® (93289; Sigma Aldrich, St Louis, MO, USA), and mRNA was extracted as per the manufacturer's protocol. The concentration of RNA in each sample was measured using a Nanodrop spectrophotometer, and 400 ng of RNA was reverse transcribed to cDNA using the high-capacity cDNA reverse transcription kit (Applied Biosystems, Foster City, CA, USA). Quantitative reverse transcription polymerase chain reaction (qRT-PCR) was performed using a 10-µL reaction mix containing 5 µL of QuantiFast SYBR Green PCR kit (Qiagen, Germany), 0.4 µL of each primer, and 4.2 µL of sample and H_2_O mix. Plates were run in an ABI 7500 Fast Real-Time PCR machine (Life Technologies, Carlsbad, CA, USA). The cycle parameters were as follows: uracil N-glycosylase activation was run for 2 minutes at 50 °C, DNA polymerase activation for 5 minutes at 95 °C, the melt cycle was run for 10 seconds at 95 °C, and the annealing–extending cycle for 30 seconds at 60 °C. A no-template control was run in each 384-well plate to confirm the absence of contamination. Each sample was normalized to reference gene *Rpl-16* and to no treatment control using a relative quantification method. All primer sequences and concentrations are outlined in Table 1.

**Table 1.** Primers used for quantitative polymerase chain reaction assays

Gene symbol	Primer sequence (5′-3′)	GenBank accession
*Rpl-19*	Fwd: gcgtgcttccttggtcttag Rev: catggaacacatccacaagc	NC_000077.7
*Amh*	Fwd: atctggctgaagtgatatgg Rev: cagggtatagcactaacagg	NM_007445.2
*Bmp15*	Fwd: atgctcaagttataccatcg Rev: gattagttcgtatgctacctg	NM_009757.4
*Gdf9*	Fwd: tcacctctacaataccgtccgg Rev: gagcaagtgttccatggcagtc	NM_008110.2
*Fshr*	Fwd: agatgaactgaatctaagcg Rev: tagacctttgtccttgagatg	NM_013523.3
*Cyp19a1 (Aromatase)*	Fwd: aacatcattctgaacatcgg Rev: agggaacattcttctcaaag	NM_007810
*Caspase3*	Fwd: cataagagcactggaatgtc Rev: gctccttttgctatgatcttc	NM_009810.3
*Bcl2*	Fwd: atgactgagtacctgaacc Rev: atatagttccacaaaggcatc	NM_009741.5
*Bax*	Fwd: cctttttgctacagggtttc Rev: atattgctgtcgagttcatc	NM_007527.3

### Histological Analysis

Follicles from all groups were mounted in 2% agar droplets before being stained with nuclear fast red stain for 5 minutes and fixed for 15 minutes in 4% paraformaldehyde (Sigma-Aldrich) and processed for standard paraffin embedding as previously described (22). Transverse 5-µm sections were taken from individual samples. Prior to staining, sections were dewaxed and rehydrated. Sections underwent antigen retrieval using citrate buffer (20 minutes). Slides were then washed with phosphate-buffered saline (PBS) and blocked (5% BSA in PBS, 1 hour at room temperature). For immunofluorescence studies, follicles were stained with either anti-Cleaved Caspase (1:500, 9664S, Cell Signaling, RRID:AB_2070042) or anti-Ki67 (1:500, ab16667, Abcam, RRID:AB_302459) primary antibodies overnight before secondary antibody Alexa 594 (A11012, RRID:AB_141359) or 488 (A11034, Invitrogen, RRID:AB_2576217) staining for 1 hour at room temperature at 1:400. The nuclear stain 4′,6-diamidino-2-phenylindole dihydrochloride (DAPI; 32670, Sigma-Aldrich) at 1:2000 in PBS was applied to all samples for 3 minutes prior to sample mounting on glass slides using ProLong Gold mounting medium (P36930, Invitrogen). Imaging was performed on a Nikon Eclipse Ti fluorescent microscope.

### Statistical Analysis

During the course of this study, images and growth data for 77 to 85 follicles, depending on treatment group, were acquired. Follicle area was measured for each follicle at the start of culture (0 hour), and every 24 hours thereafter, up to 96 hours. Relative area was calculated for each follicle between 0 hour (baseline) and 24 hours, 48 hours, 72 hours, and 96 hours (area time x/area time 0) and cumulative growth curves generated. Follicles with a central spherical oocyte and intact layer of GCs were included in the analysis. Follicles were excluded from analysis if, at the start of culture, their oocyte was misshapen or extruded from the follicle, if the follicle was atretic (darkened GCs), or if the basal lamina was damaged with the oocyte being subsequently extruded from the follicle during culture. For follicles that showed signs of atresia during the culture period, the time point was noted for survival data and follicles were excluded from further longitudinal analysis. The images provided an extended dataset for analysis of developmental and morphological features such as antrum formation or for comparing development of follicles of differing sizes under various treatments. The average change in follicle area over all follicles was calculated for each treatment group to give a single summary measure and a mixed-effects analysis was used to compare the means of the summary measures.

Following culture, at least 5 follicles were grouped together and snap frozen for qPCR analysis. The relative expression of each gene was normalized to expression of *Rpl-19* and the results were log_2_ transformed and expressed mean ± SEM fold-change gene expression relative to the control group at 24 hours. Follicle area, survival time and qPCR data were analyzed using 2-way analysis of variance (ANOVA) with the Tukey post hoc test, while the survival rate was analyzed using a 1-way ANOVA with Dunnett's post hoc test, using GraphPad Prism 9.3.1. No adjustments were made to the data for multiple comparison tests. In all experiments, a probability value of less than *P* < .05 was considered to be statistically significant. Technical replicates are represented as N, while biological replicates are represented as n.

## Results

### Classification of Isolated Preantral Follicles and Response to FSH in Culture

The follicles that were manually isolated from ovaries of 3- to 5-week-old mice in these studies ranged between 70- and 150-µm in diameter. This age range was chosen as mice were prepubertal and yet to undergo synchronized gonadotropin hormone priming that results in first estrus. The follicles were cleanly isolated, with only a few theca cells adherent to the basal lamina (Fig. S1A-Aiv (44)). Based on measurements of follicles from the micrographs taken on day 0, 70-µm diameter follicles had at least 1 full layer of cuboidal GCs, equivalent to a primary follicle beginning to form a second layer. Follicles with diameters of 100 µm approximate to the secondary stage with 2 complete layers of GCs. Pre-antral follicles with diameters greater than 100 µm exhibited more than 2 GC layers. Follicles with indistinct oocytes (Fig. S1B-Biv (44)), ruptured basement membranes (Fig. S1C-Civ (44)), GC atresia (Fig. S1D-Div (44)), or cumulus cell complexes (Fig. S1E-Eiv (44)) were excluded from analysis.

### Follicle Growth, Morphology, and Survival Are Differentially Regulated by FSH Glycoforms

Follicle size did not affect the response of pre-antral follicles to FSH glycoforms (Fig. S2 (44)). When stratified by size, similar increases in FSH glycoform-dependent follicle growth were observed at 70 to 100μm (Fig. S2A (44)), 101 to 120μm (Fig. S2B (44)) 121 to 140μm (Fig. S2C (44)), and 141 to 180μm (Fig. S2D (44)). Therefore, for subsequent experiments, pre-antral follicles were pooled for data analysis.

**Figure 1.**
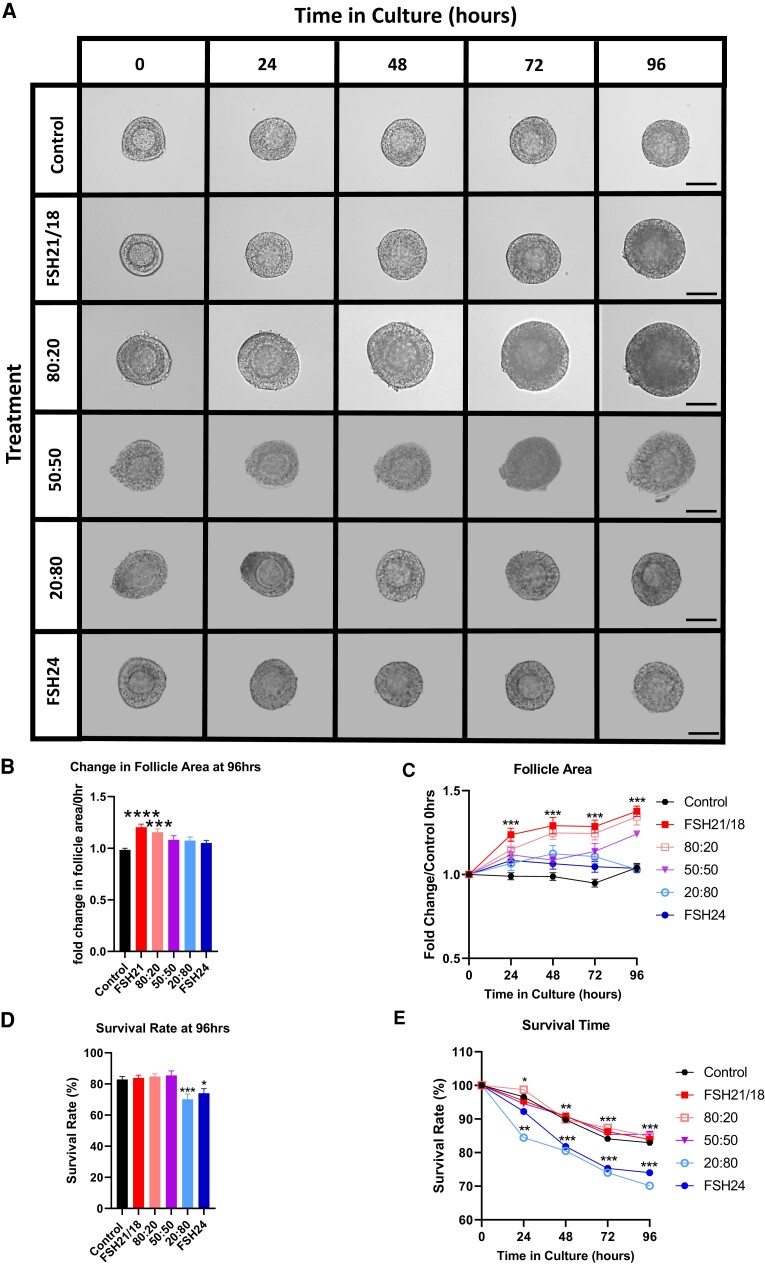
FSH glycoforms differentially regulate follicle growth and survival. (A) Representative morphology of preantral follicles cultured for 0 to 96 hours, in response to differential FSH glycoform treatment. Scale bars = 50 µm. (B) The average change in follicle area over 11 follicles was calculated from treatment group to give a single summary measure and a mixed-effect analysis was used to compare the means of the summary measures. (C) Individual pre-antral follicles were cultured ±FSH21/18, 80:20 FSH21/18:FSH24, 50:50 FSH21/18:FSH24, 20:80 FSH21/18:FSH24, and FSH24 for 0 to 96 hours and follicle area measured daily (n = 77-85 follicles analyzed over 11 independent experiments). The relative area of follicles in different treatments (area at time x/area at time 0, where time x = 24, 48, 72, or 96 hours) was compared at each time point using 2-way ANOVA. *P < .05, **P < .01, ***P < .001, ****P < .0001. Values are mean and SEM. (D) Survival rate following 96-hour culture period, with micrographs from (A) analyzed for follicle survival. (E) The percentage of live follicles as assessed at 24-hour intervals during the 96-hour culture period.

To determine the effect of FSH glycoforms on follicle growth and survival, isolated follicles were treated with ±10 ng/mL FSH21/18 or FSH24, and ratios of FSH21/18:24 to recapitulate the previously described aging-related modulation in FSH glycoforms observed with aging- reproductive prime (80:20 FSH21/18:FSH24), perimenopause (50:50 FSH21/18:FSH24), and menopausal (20:80 FSH21/18:FSH24). Follicle growth was monitored via daily micrographs for up to 96 hours (Fig. 1A), and changes in follicle health (darkening of GCs, basement membrane rupture and oocyte) recorded. FSH21/18 and FSH21/18:FSH24 at 80:20 resulted in significant increases in follicle area over the 96-hour treatment period (Fig. 1B). Treatment of follicles with FSH21/18 promoted rapid follicular growth, with 1.24-fold increase observed at 24 hours compared with control and continued increase in growth for the 96-hour time period. The 80:20 ratio of FSH21/18:FSH24 also increased follicle growth by 1.15-fold at 24 hours compared with no treatment controls (0 ng/mL FSH; Fig. 1C). Treatment of follicles with 50:50 FSH21/18:FSH24 displayed slower growth kinetics than FSH21/18 dominant treatment conditions, with follicle growth significantly increased by 1.14-fold at 72 hours of culture compared with the no treatment control, FSH24, and FSH21/18:FSH24 20:80 (*P* < .05, Fig. 1C). In contrast to the FSH21/18 dominant treatments, follicles cultured in FSH24 dominant conditions, FSH21/18:FSH24 20:80 and FSH24-alone groups, failed to elicit a significant increase in follicle area at any time point, suggesting that FSH21/18 promotes follicle growth, and declining amounts of FSH21/18 and increasing FSH24 are detrimental to follicle growth.

Next, morphological analysis of follicle micrographs was carried out and numerically analyzed to generate follicle survival curves as a function of time. Follicles treated with FSH24 or 20:80 FSH21/18:FSH24 showed increased follicle atresia by 96 hours in culture compared with all other treatments (Fig. 1D). When analyzed by day for follicle atresia, a significant decrease in follicle survival was observed by 24-hour culture with 20:80 FSH21/18:FSH24 treatment, and by 48 hours with FSH24 (Fig. 1E). This contrasted with no significant changes from control for follicles cultured in FSH21/18, 80:20 FSH21/18:FSH24, and 50:50 FSH21/18:FSH24. Taken together, these data indicate that FSH24 promotes follicle atresia, while FSH21/18 sustains follicle growth and survival.

### Granulosa Cell Apoptosis and Proliferation Are Regulated in a FSH Glycoform-Dependent Manner

**Figure 2.**
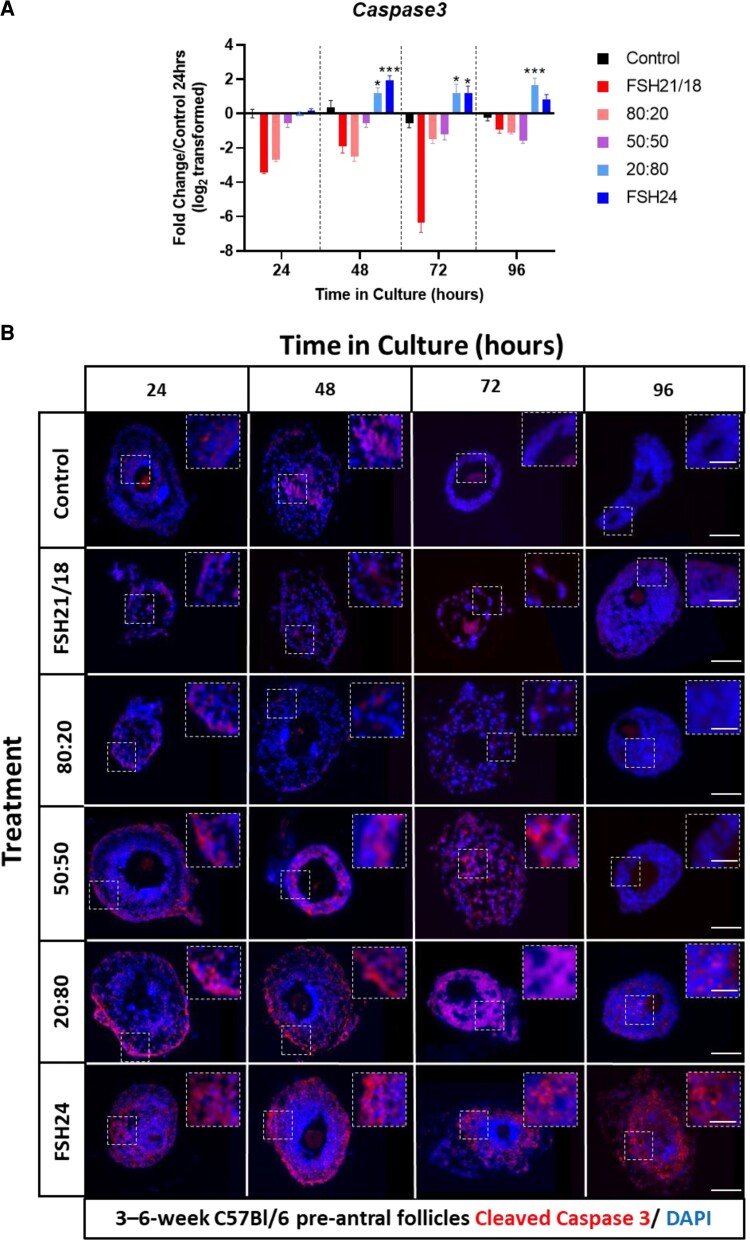
Expression of apoptosis-related Caspase3 is increased in follicles treated with FSH24 dominant conditions. (A) Individual pre-antral follicles were cultured ±FSH21/18, 80:20 FSH21/18:FSH24, 50:50 FSH21/18:FSH24, 20:80 FSH21/18:FSH24, and FSH24 for 0 to 96 hours. At the end of the treatment, 6 to 10 follicles from 4 to 6 mice in each technical replicate were pooled and snap-frozen and analyzed by quantitative RT-PCR for *Caspase3*, n = 3. Data are represented as fold change relative to untreated follicles at 24 hours (value = 0) and analyzed using 2-way ANOVA *P < .05, **P < .01, ***P < .001. Values are mean and SEM. (B) Representative images of follicles cultured ±FSH glycoforms as outlined in (A). At the end of the culture period, individual follicles were fixed, paraffin embedded and sectioned, before staining for CLEAVED CASPASE 3 (65) and counterstained with nuclear stain, DAPI (blue). N = 3 for each time point. Scale bar = 50 μm. Insert scale bar = 25 μm.

To understand how FSH glycoforms mediated these differential effects on follicle growth and survival, we next investigated how FSH glycoforms modulated markers of apoptosis and proliferation. The expression of the apoptosis-related gene, *Caspase3*, a protease which cleaves cellular targets and executes cell death, increased in a time-dependent manner in follicles treated with either 20:80 FSH21/18:FSH24 (1.02-3.16-fold change; *P* < .001) or FSH24 (1.15-3.79-fold change; *P* < .05) from 48 hours of culture. Indicating that FSH24 is detrimental to pre-antral follicle survival in this culture system (Fig. 2A). Conversely, treatment of pre-antral follicles with either FSH21/18, 50:50 FSH21/18:FSH24, or 80:20 FSH21/18:24 resulted in decreased *Caspase 3* expression across all time points assessed, supporting a potential role of FSH21/18 as a survival factor.

Further to gene expression, the protein expression of CLEAVED CASPASE-3 was investigated. Immunohistochemical analysis revealed that the protein expression of CLEAVED CASPASE-3 mirrored the gene expression data, with expression highest in 20:80 FSH21/18:FSH24- and FSH24-treated follicles, and lowest in FSH21/18, 50:50 FSH21/18:FSH25, and 80:20 FSH21/18:FSH24 treatment groups. In FSH24 dominant treatment groups, analysis of CLEAVED CASPASE-3 spatial staining pattern suggested initial staining was localized to the basement membrane, before becoming more centrally GC localized from 48 hours of culture, potentially explaining the increased basement membrane rupture and oocyte extrusion observed at the same time points (Fig. 2B). In contrast, CLEAVED CASPASE-3 expression in FSH21/18 dominant and 50:50 FSH21/18:FSH24 treatment groups was minimal and present in a mainly diffuse pattern through the GCs.

**Figure 3.**
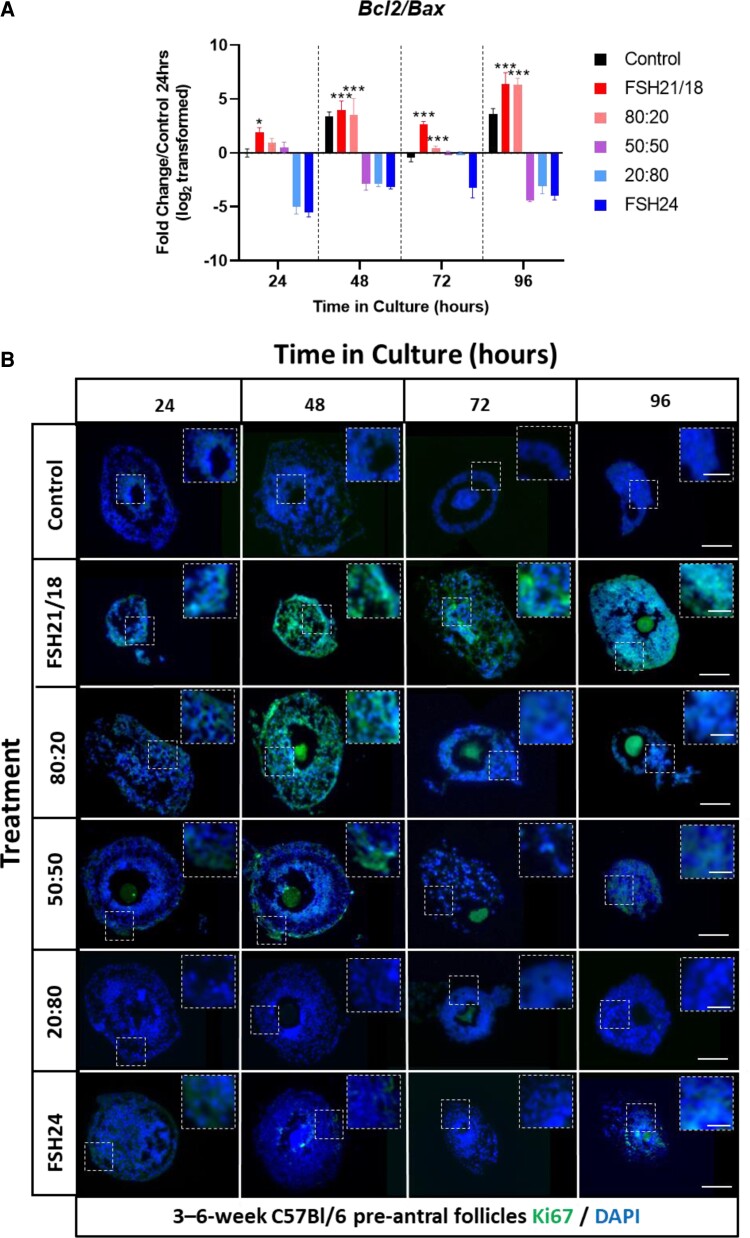
FSH21/18 promotes the expression of proliferation and anti-apoptotic markers. (A) Individual pre-antral follicles were cultured ±FSH21/18, 80:20 FSH21/18:FSH24, 50:50 FSH21/18:FSH24, 20:80 FSH21/18:FSH24, and FSH24 for 0 to 96 hours. Groups of 6 to 10 follicles from 4 to 6 mice in each group were pooled, snap-frozen, and analyzed by quantitative RT-PCR, n = 3. Data were expressed as fold change relative to control untreated follicles at 24 hours (value = 0). Data were statistically analyzed by 2-way ANOVA *P < 0.05, **P < .01, ***P < .001. Values are mean and SEM. (B) Representative images of follicles cultured for 0 to 96 hours ±FSH glycoforms as outlined in (A). Follicles were fixed, paraffin embedded, and sectioned, and stained for the cell proliferation marker Ki67 (green) and counterstained with the nuclear marker, DAPI (blue) and analyzed by fluorescent microscopy. N = 3 for each time point. Scale bar = 50 μm. Insert scale bar = 25 μm.

Next to investigate the effects of FSH glycoforms on GC proliferation, the gene expression of *Bcl2/Bax* (high *Bcl2* to *Bax* ratio is indicative of resistance to apoptosis (45)) and Ki67 at a protein level were analyzed. FSH21/18 and 80:20 FSH21/18:FSH24 promoted proliferation at all time points assessed, as evidenced by the increase in *Bcl2/Bax* expression (*P* < .05; Fig. 3A). In pre-antral follicles treated with FSH21/18 and 80:20 FSH21/18:FSH24, a biphasic expression pattern of *Bcl2/Bax* was observed, with an initial increase in *Bcl2/Bax* up to 48 hours (3.72-15.81-fold change and 1.94-11.74-fold change, *P* < .001, Fig. 3A), decreasing at 72 hours (15.81-6.30 and 11.74-8.07-fold change, *P* < .001, Fig. 3A), and further increasing at 96 hours (6.30-18.96 and 8.07-9.31-fold change, *P* < .001; Fig. 3A). In contrast, an inhibition in *Bcl2/Bax* expression was observed in pre-antral follicles treated with either 50:50 FSH21/18:24, 20:80 FSH21/18:24, or FSH24 (Fig. 3A), mirroring the increase in apoptosis previously observed.

Immunofluorescent analysis of Ki67 expression revealed FSH21/18 dominant treatment groups enhanced Ki67 expression, indicating that FSH21/18 promotes GC proliferation (Fig. 3B), consistent with the follicle area data. Conversely, little Ki67 expression was found in pre-antral follicles treated with predominantly FSH24 (Fig. 3B). Taken together these data indicate that FSH21/18 promotes GC proliferation, resulting in increased follicle area.

### FSH Receptor, Steroidogenesis and Folliculogenesis Associated Gene Expression are Altered With Differential FSH Glycoform Culture

**Figure 4.**
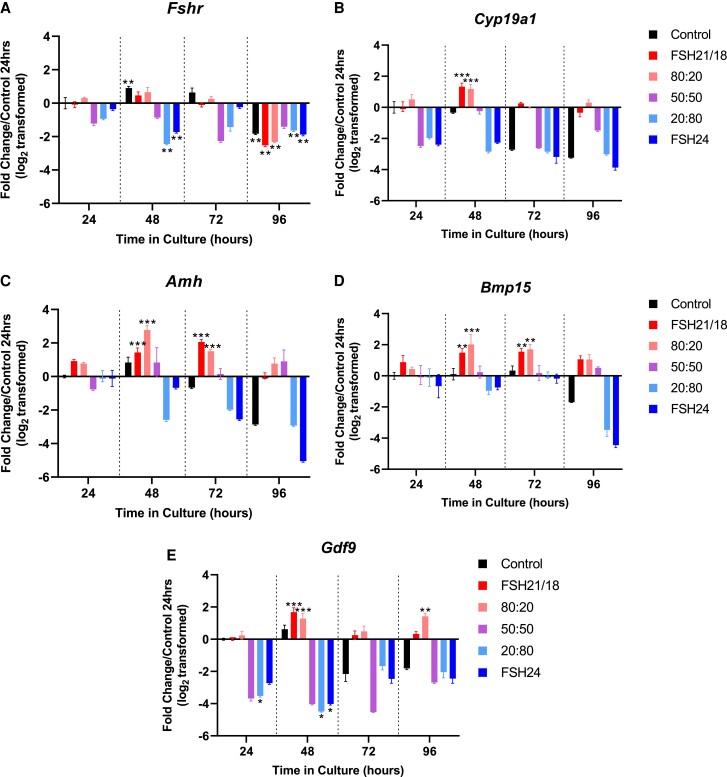
FSH glycoforms differentially regulate the expression of key ovarian follicle and FSH responsive genes. A 0- to 96-hour time course analysis of (A) FSH receptor (*Fshr*), (B) Aromatase (*Cyp19a1*), (C) anti-Mullerian hormone (*Amh*), (D) bone morphogenic protein 15 (*Bmp15*), (E) growth differentiation factor-9 (*Gdf9*) expression following treatment ±10 ng/mL FSH21/18 80:20 FSH21/18:FSH24, 50:50 FSH21/18:FSH24, 20:80 FSH21/18:FSH24, and FSH24. Groups of 6 to 10 follicles from 4 to 6 mice in each group were pooled and snap-frozen and analyzed by quantitative RT-PCR, n = 3. Data are expressed as fold change relative to untreated follicles at 24 hours (value = 0), and statistically analyzed using 2-way ANOVA *P < .05, **P < .01, ***P < .001. Each data point represents mean −/+ SEM.

To understand how FSH glycoforms modulate key FSH target genes and pathways and well-established markers of follicle health and development, the expression of *Fshr*, *Cyp19a1*, *Amh*, *Bmp-15*, and *Gdf9* was analyzed via qPCR. FSH21/18 and 80:20 FSH21/18:24 had little effect on *Fshr* expression at all time points compared with control (Fig. 4A). Interestingly, follicles cultured in 50:50 FSH21/18:FSH24, 20:80 FSH21/18:FSH24, or FSH24 alone decreased *Fshr* expression at all analyzed time points.

Analysis of *Cyp19a1* expression revealed FSH21/18 and FSH21/18:24 at 80:20 follicles increased *Cyp19a1* gene expression by 48 hours (2.51- and 2.29-fold change vs control, *P* < .001; Fig. 4B), decreasing thereafter. Moreover, follicles cultured in the presence of FSH21/18:24 at 50:50, 20:80, and FSH24 had lower aromatase expression than control, at 24- and 48-hour time points (*P* < .05; Fig. 4B), but mirrored control at 72 and 96 hours.

The expression of anti-Müllerian hormone (*Amh*)is initiated in growing follicles, persisting into antral stages of development until dominant follicle selection (46, 47). *Amh* is an important inhibitory factor, regulating sensitivity to FSH. Follicles cultured in the presence of FSH21/18 and 80:20 FSH21/18:24 increased *Amh* gene expression at all time points until 96 hours, when a decrease was observed (Fig. 4C). Follicles treated with 50:50 had little effect on *Amh* expression compared with control at all time points. Interestingly, a trend for decreased *Amh* expression was found at all time points in follicles cultured in the presence of either 20:80 FSH21/18:24 or FSH24 (Fig. 4C). Together these data indicate that FSH21/18 dominant conditions enhance *Fshr* and *Cyp19a1* expression when *Amh* expression is highest, indicating a potential mechanism to overcome the inhibitory actions of *Amh* on follicle growth and survival.

The oocyte-derived TGFb family members, bone morphogenetic protein 15 (*Bmp15*), and *Gdf9* are trophic paracrine factors, with key roles in promoting GC functions. Interestingly, follicles cultured in the presence of FSH21/18 or 80:20 FSH21/18:FSH24 had an increase in *Bmp15* gene expression from the earlier 24-hour and 48-hour time points, with a decrease in bmp15 expression thereafter (Fig. 4D). While expression decreased in these groups from 48 to 96 hours, this remained significantly higher than the control (*P* < .05; Fig. 4D). *Bmp15* expression remained unchanged and comparable with the 24-hour control in 20:80 FSH21/18:24- and FSH24-treated follicles, until decreasing in expression at 96 hours (*P* > .05; Fig. 4D), while follicles in the 50:50 showed little change in *Bmp15* expression at all time points assessed.

In line with the observation with *Bmp15*, *Gdf9* expression was increased in follicles treated with FSH21/18 (0.98-3.19-fold change; *P* < .001) and 80:20 FSH21/18:FSH24 (1.18-1.2.42-fold change; *P* < .001; Fig. 4E) following 24 hours of culture, with expression decreasing thereafter (Fig. 4E). Contrastingly, follicles cultured in the presence of FSH24, 20:80 FSH21/18:FSH24, or 50:50 FSH21/18:FSH24 displayed decreased *Gdf9* expression at 24, 48 and 72 hours of culture compared with control (*P* < .05; Fig. 4E). Together, these data support the trophic role of FSH21/18 as a survival factor, with that of FSH24 as promoting follicle atresia.

### FSH21/18 and FSH24 Mediate Their Actions via Differential Signaling Pathways

While it is widely agreed that the effects of FSH and its differential functions are mediated by the activation of the G*α*s/cAMP/PKA pathway, it is becoming increasingly apparent that this is an oversimplified model of FSH/FSHR actions. Therefore, isolated follicles were treated with inhibitors for PKA, ERK or PI3K signaling pathways, and assessed for their ability to respond to different FSH glycoforms.

**Figure 5.**
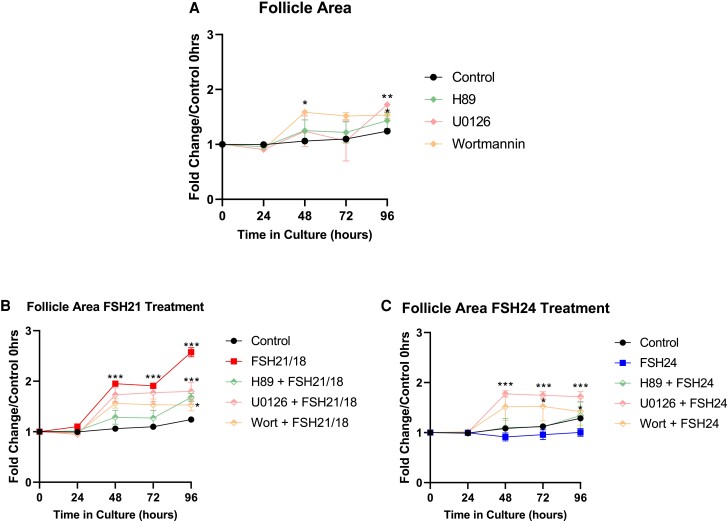
FSH glycoform specific effects on follicle growth are mediated via differential signal pathways. (A) Control responses of individual follicles to 1 mM H89 (cAMP inhibitor), 1 mM U0126 (MAPK inhibitor), and 100 nM wortmannin (Pi3K inhibitor) over the 96-hour culture period (n = 9-36 individual follicles assessed/treatment group, n = 3). (B) Response of individual follicles to FSH21/18 and pathway inhibitors in combination with FSH21/18 (n = 9-36). (C) Growth response curves of individual follicles FSH24 and pathway inhibitors in combination with FSH24 (9-29 follicles per treatment group, n = 3). The relative area of follicles in different treatments (area at time x/area at time 0, where time x = 24, 48, 72 or 96 hours) was compared at each time point using 2-way ANOVA. *P < .05, **P < .01, ***P < .001. Values are mean ± SEM.

First, as a control, the effect of inhibiting each pathway was assessed. Inhibition of PKA by H89 had minimal impact on follicle growth (Fig. 5A). Interestingly, inhibition of the ERK/MAPK pathway with U0126 had no effect on follicle growth, until 96 hours of culture, where follicle area was enhanced by 1.7-fold compared with control (*P* < .01; Fig. 5A). Inhibition of the PI3K pathway with wortmannin, positively impacted follicle growth, with increase in follicle area observed from 48 hours of culture (*P* < .05; Fig. 5A).

As a graded response was observed with ratios of FSH21/18:FSH24 in terms of FSH21/18 dominant conditions promoting follicle growth and survival and FSH24 dominant follicle atresia to dissect the signaling pathways mediating these effects, follicles were treated with FSH21/18 or FSH24 alone. In concordance with previous observations, treatment with 10 ng/mL FSH21/18 resulted in an increase in follicle area (*P* < .0001; Fig. 5B). Interestingly, cotreatment of FSH21/18 with any of the inhibitors dampened the trophic effects of FSH21/18, suggesting an FSH21/18 activation of and dependency on multiple signal pathways. Inhibition of PKA (H89 treatment) had the most pronounced inhibition on FSH21/18 action, inhibiting FSH21/18 increased follicle area until 96 hours of treatment (*P* < .05; Fig. 5B). Inhibition of the ERK/MAPK (via U0126) and PI3K (via wortmannin) pathways had less of an effect on FSH21/18-dependent follicle growth (Fig. 5B). In the presence of either U0126 or Wortmannin, FSH21/18 still elicited a significant increase in follicle area compared with control at 48 hours (*P* < .001; Fig. 5B), but plateauing thereafter. These results suggest that while the effects of FSH21/18 on follicle growth may primarily be mediated via the PKA pathway, roles for both the ERK-MAPK and PI3K pathways in eliciting a full FSH21/18-dependent response may exist.

In concordance with previous results, FSH24 had a negative effect on follicle growth. Surprisingly, cotreatment of FSH24 with H89 resulted in a modest increase in follicle area, which was comparable with that of the untreated control (Fig. 5C). Moreover, inhibition of ERK-MAPK resulted in an increase in FSH24-dependent follicle growth, mimicking the growth curves observed with FSH21/18 and U0126 cotreatment. Inhibition of PI3K with wortmannin resulted in increased FSH24-dependent follicle growth; however, this was comparable with the timing and magnitude observed in control conditions with wortmannin treatment alone (Fig. 5A), indicating that this was likely the effect of wortmannin cotreatment. These data indicate that FSH21/18 and FSH24 may display bias in signal pathway activation and that this in turn mediates the differential effects of FSH glycoforms on follicle growth.

### Inhibition of PKA, ERK, and PI3K Pathways Differentially Affect Apoptotic Gene Expression

**Figure 6.**
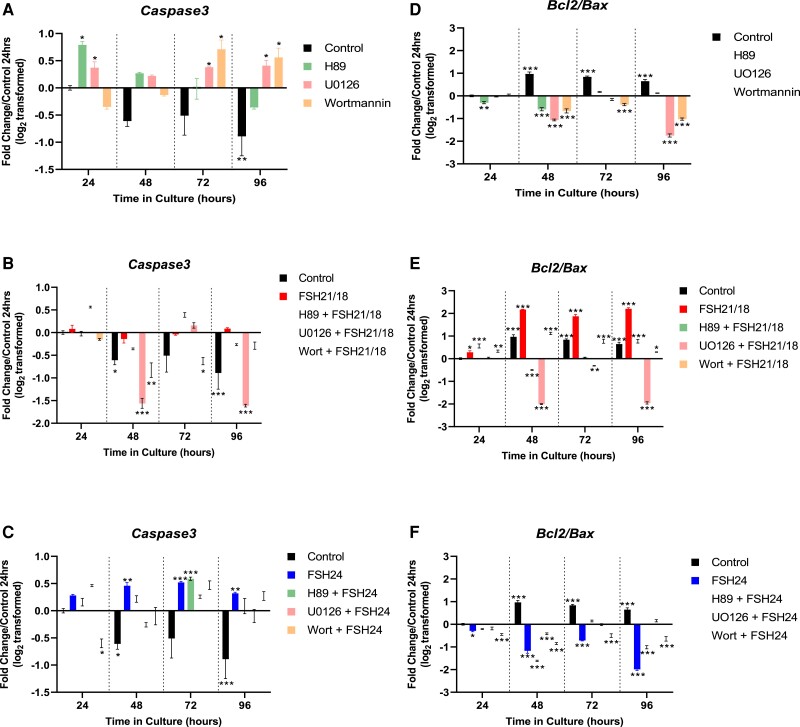
FSH glycoforms mediate their pro and anti-apoptotic effects via differential signal pathways. Time-dependent effect of (A) H89, U0126 and wortmannin alone (B) cotreatment with FSH21/18 and (C) cotreatment with FSH24 on *Caspase3* gene expression. Effect of (D) H89, U0126 and wortmannin alone (E) cotreatment with FSH21/18 and (F) cotreatment with FSH24 on *Bcl2/Bax* ratio. Each data point is representative of 6 to 10 follicles pooled from 4 to 6 mice/group, n = 3. Data are represented as fold change relative to untreated follicles at 24 hours (value = 0), and analyzed using 2-way ANOVA *P < .05, **P < .01, ***P < .001. Values are mean ± SEM.

Next, the role of differential FSH glycoform-utilized signal pathways on apoptotic and proliferative gene markers were assessed. In control, inhibitor-treated follicles, *Caspase3* gene expression was differentially regulated by the 3 signal pathway inhibitors (Fig. 6A). Treatment with H89 alone increased *Caspase3* at 24-hour culture, with expression decreasing from 48 to 96 hours of culture (*P* < .05; Fig. 6A). Treatment with U0126 induced a significant increase in *Caspase3* expression at all time points (*P* < .05; Fig. 6A), whereas wortmannin treatment increased *Caspase 3* expression from 48 to 96 hours (*P* < .05; Fig. 6A).

Treatment with FSH21/18 alone reduced *Caspase3* gene expression at all time points (Fig. 6B). Interestingly, cotreatment with H89 had little effect on *Caspase3* expression at any time point, despite displaying the most prominent effects on FSH21/18-mediated follicle growth (Fig. 6B). U0126 cotreatment resulted in a marked further decrease in FSH21/18-dependent *Caspase3* expression at 48 and 96 hours of culture (*P* < .001; Fig. 6B). Moreover, FSH21/18 cotreatment overcame the increase in *Caspase3* observed with the inhibition of PI3K alone (*P* < .05; Fig. 6B). These indicate that FSH21/18-dependent effects on follicle growth and survival are at least in part mediated by anti-apoptotic effects.

As expected, treatment of pre-antral follicles with FSH24 alone increased *Caspase3* expression at all time points (*P* < .001; Fig. 6C), as seen in earlier experiments (Fig. 2A). Interestingly, cotreatment with all inhibitors resulted in a decrease in FSH24-dependent *Caspase3* expression, with inhibition of ERK/MAPK and PI3K having the most profound effect. These data suggest that the enhanced FSH24-dependent follicle growth observed in the presence of H89, U0126, and potentially wortmannin may be mediated via decrease in the activation of apoptotic pathways.

Next, we investigated the effect of inhibiting different signaling pathways on anti-apoptotic markers, *Bcl2/Bax*. Inhibition of PKA via H89 resulted in initial decreased *Bcl2/Bax* at 24 and 48 hours compared with control (*P* < .01; Fig. 6D), with an increase at the 72- and 96-hour time points. Inhibition of the ERK and PI3K pathways significantly decreased *Bcl2/Bax* from 48 hours (*P* < .001; Fig. 6D), suggesting inhibition of these pathways promoted apoptosis.

Treatment of follicles with FSH21/18 alone, resulted in an increase in the anti-apoptotic gene expression of *Bcl2/Bax*, as previously described (Fig. 6E). Inhibition of PKA had little effect on FSH21/18-dependent Bcl2/Bax expression; however, a decrease was found at 48 and 72 hours in cotreated follicles (*P* < .001; Fig. 6E). Inhibition of the ERK pathway via U0126 was sufficient to block the anti-apoptotic effect of FSH21/18, where decreases in *Bcl2/Bax* ratios were found from 48 to 96 hours (*P* < .01; Fig. 6D). Interestingly, inhibition of the PI3K pathway with wortmannin treatment, partially decreased the ability of FSH21/18 to modulate the expression of *Bcl2/Bax* compared with FSH21/18 treatment alone, (*P* < .001; Fig. 6D). These data suggest that multiple signaling pathways mediate FSH21/18-dependent follicle growth.

Treatment with FSH24, resulted in decreased in *Bcl2/Bax*. Although inhibition of PI3K and PKA pathways had little effect on FSH24-dependent actions, inhibition of ERK-MAPK reduced the *Bcl2/Bax* (*P* < .05; Fig. 6F). This suggests that FSH24 modulation of the MAPK pathway may be an important mechanism by which pro-apoptotic signals are activated by FSH24. Follicle survival was differentially affected by signaling pathway inhibitors (Fig. S3A (44)) and cotreatment of FSH21/18 (Fig. S3B (44)) and FSH24 (Fig. S3C (44)) with U0126 had the greatest decrease in follicle survival, while cotreatment with wortmannin had the least effect (Fig. S3A (44)). Cotreatment of FSH21/18 and H89 resulted in decreased follicle survival compared with FSH21/18 treatment alone (Fig. S3B (44)). When analyzed by day for follicle atresia, no significant effect on day was found (Fig. S3D-F (44)).

### Steroidogenesis Associated Gene Expression Is Altered With Differential Pathway Inhibitors

**Figure 7.**
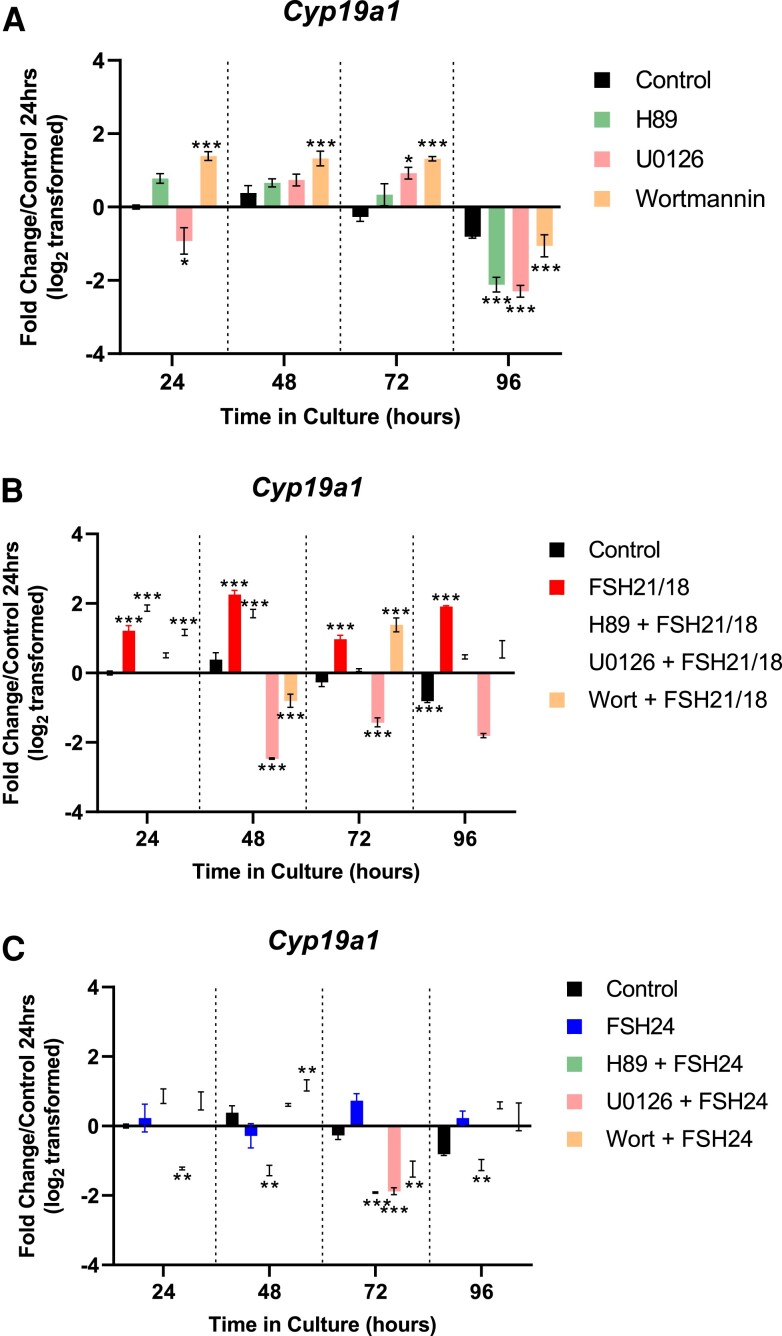
FSH glycoforms modulate aromatase gene expression via differential signal pathways. Time-dependent effect of (A) H89, U0126 and wortmannin alone, (B) cotreatment with FSH21/18, and (C) cotreatment with FSH24 on *CYP19a1* gene expression. Each independent experiment is representative of 6 to 10 pooled follicles from 4 to 6 mice, n = 3. Data are represented as fold change relative to untreated follicles at 24 hours (value = 0), and analyzed using 2-way ANOVA *P < .05, **P < .01, ***P < .001. Values are mean −/+ SEM.

Lastly, we investigated what effects inhibition of different signaling pathways had on aromatase gene expression. Inhibition of PKA signaling had no effect on *Cyp19a1* gene expression from 24 to 72 hours, following which a decrease was observed at 96 hours (*P* < .001; Fig. 7A). When follicles were treated with the MAPK inhibitor U0126, an initial decrease at 24 hours (*P* < .05) was followed by increases at 48 and 72 hours (*P* < .05), and a decrease at 96 hours (*P* < .001; Fig. 7A). Lastly, wortmannin treatment increased *Cyp19a1* expression from 24 to 72 hours (*P* < .001), before significantly decreasing at 96 hours (*P* < .001; Fig. 7A), suggesting that inhibition of Pi3K signaling may have a positive effect on steroidogenesis.

Cotreatment of FSH21/18 with signaling pathway inhibitors resulted in differential *Cyp19a1* gene expression. As seen previously, treatment with FSH21/18 alone resulted in an increase in *Cyp19a1* expression at all time points (*P* < .0001; Fig. 7B). Interestingly, cotreatment of FSH21/18 and H89 resulted in an increase in *Cyp19a1* at 24 and 48 hours (*P* < .001; Fig. 7B), with inhibition thereafter. No change in *Cyp19a1* expression was found at 24 hours following cotreatment with U0126 and FSH21/18; however, from 48 to 96 hours there was a significant decrease in *Cyp19a1* expression (*P* < .001; Fig. 7B). Follicles cotreated with wortmannin had little effect at the 24-hour time point, but abrogated FSH21/18-dependent *Cyp19a1* expression at 48- and 96-hour time points (*P* < .001; Fig. 7B).

**Figure 8.**
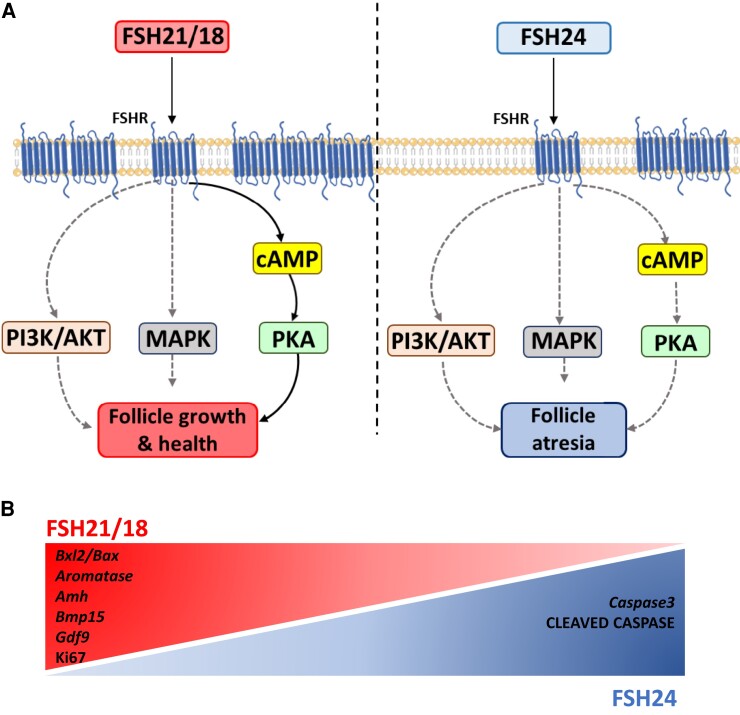
Schematic model of FSH21/18 and FSH24 signaling in pre-antral follicles. (A) FSH21/18 and FSH24 G protein–dependent and –independent pathway activation through receptor–G signaling protein interactions and receptor–adapter/scaffolding protein interactions. Dashed lines represent currently hypothesized/unresolved mechanisms of pathway activation, and spear-headed arrows are activated pathways. (B) Gradients indicating increases in genes and proteins in response to FSH21/18, FSH24 and ratios thereof. The Figure was partly generated using Servier Medical Art, provided by Servier, licensed under a Creative Commons Attribution 3.0 unported license.

Similarly to FSH21/18 cotreatment, differential expression of *Cyp19a1* was found following FSH24 cotreatment. H89 cotreatment with FSH24 resulted in a significant decrease in FSH24-dependent Cyp19a1 expression by 48 hours and sustained at all time points thereafter (*P* < .01; Fig. 7C). Furthermore, cotreatment with U0126 resulted in a biphasic response, with a decrease in FSH24-dependent *Cyp19a1* expression at 24 and 72 hours with increases at 48 and 96 hours (*P* < .01; Fig. 7C). Lastly, wortmannin increased *Cyp19a1* expression at 24, 48, and 96 hours; however, a decrease, as with the other cotreatments, was found at 72 hours (*P* < .001; Fig. 7C). Taken together these data suggest that FSH glycoforms signal via alternative mechanisms and have prominent roles in proliferation and apoptotic signaling (Fig. 8).

## Discussion

FSH is a key regulator of ovarian function, which undergoes age-dependent modulation in its glycosylation. This study investigated how FSH21/18, which predominates in women of reproductive prime with intact ovulatory menstrual cycles, and FSH24, which increases with age and predominates in perimenopausal/menopausal women and ratios thereof, modulated murine preantral follicle growth and survival. Our data support that FSH21/18 enhances follicle growth and survival through enhancement of pathways that promote cell proliferation and modulation of key factors that support follicle development. This contrasts with FSH24, which promotes follicle atresia through promoting apoptosis. Furthermore, our data suggest that this may in part be mediated via ligand bias and utilization of differential signal pathways by FSH glycoforms (Fig. 8).

Previous studies investigating the effects of partially glycosylated FSH21/18 and fully glycosylated FSH24 glycoform preparations have predominately utilized acute measurements in heterologous cell lines expressing FSHR, isolated GCs, and in vivo and ex vivo ovaries (43, 41) This study provides important next steps in understanding the effects of FSH glycoforms on longer-term pre-antral follicle cultures, analyzing the effects of FSH glycoforms when follicles become sensitive to FSH. Moreover, this study presents the first ratiometric analysis of FSH21/18:FSH24, mimicking the reported aging-dependent changes and showing titratable effectiveness of FSH21/18 in promoting follicle growth and survival with increasing in FSH24. Our findings not only support previous findings that FSH21/18 is a more potent activator of FSHR-dependent pathways, promoting specific gene expression signatures. Our data provide the next steps in understanding the physiological roles of this, with distinct FSH21/18 pathway activation ultimately supporting pre-antral follicle growth and survival in more chronic follicle cultures.

FSH glycoforms differentially affected follicle survival and apoptosis. Indeed, FSH24 or FSH24-predominant FSH21/18:FSH24 combinations, such as 20:80, mimicking previously reported perimenopause/menopausal ratios, resulted in decreased survival and increased *Caspase3* expression. These data support previous studies, where FSH24 was shown to increase *Caspase3* in acutely treated isolated murine follicles (48). Moreover, FSH21/18 and ratios of FSH21/18:FSH24 mimicking reproductive prime appear to support FSH21/18 being a trophic survival factor, promoting follicle survival permitting follicles to survive. Indeed, previous studies have also shown the importance of recombinant FSH preparation on pre-antral follicle growth (22, 49, 50). As FSH24 increases with age, with potential to promote follicle atresia, it may in part contribute to the accelerated follicle depletion observed with aging (51, 52). Furthermore, the differences in FSH target gene expression (steroidogenic enzymes) are in concordance with previous in vitro studies in cultured human GCs (53) and in vivo studies (41, 43).

Our data suggest that FSH21/18, FSH24 and the ratios of FSH21/18:FSH24 have distinct gene expression signatures. The relationship between FSH and AMH is complex, with FSH and AMH negatively correlated, where high levels of FSH, as observed with aging, indicates low AMH and subsequent low ovarian reserve (54). AMH expression has been shown to initiate at follicle recruitment, where it inhibits the cyclical process of follicular recruitment by determining FSH threshold levels (47). Therefore, the initial increase in *Amh* with FSH21/18 treatment may be a regulatory recruitment process in the pre-antral follicles. Similarly, stimulation of proliferation was observed previously in follicles cultured in the presence of *Bmp15* and *Gdf9* together suggesting an increase in cell turnover may be induced as a self-regulatory mechanism to prevent excessive follicle growth (55).

The PKA, MAPK, and PI3K pathways are involved in regulation of follicle development. MAPK via extracellular signal-regulated kinase 1 (ERK1) and ERK2 phosphorylates MYC protein that modulates growth, proliferation and survival (56). Moreover, MAPK/ERK signaling has also been suggested to be important in FSH mediated maturation of preovulatory follicles (57). Importantly, 2 of the main signaling pathways activated by FSH via PKA are the PI3K and ERK pathways (28). Moreover, the canonical PKA-dependent pathway and MAPK pathways are both required for full FSH biological activity, while the PI3K pathway plays a pivotal role in FSH-induced activity including gene transcription, proliferation, and protein translation (41). Herein, we highlight, using pharmacological inhibition of these pathways, the loss of FSH21/18 induced proliferation in the presence wortmannin (PI3K inhibitor) and U0126 (MAPK inhibitor). Our data support our previous observations in cultured ovarian wedge sections (41). Additionally, FSH21/18-induced follicle growth was inhibited when PKA was blocked, highlighting that FSH21/18 does in fact signal primarily via the Gαs/AC/cAMP/PKA pathway. These data are consistent with published observations by us, and others using heterologous cell lines primary GCs and acutely treated isolated murine follicles, with FSH21/18 efficiently increasing cAMP production, while FSH24 weakly increases cAMP (40, 48, 58). Interestingly, PKA inhibition had no effect on follicle growth when treated in combination with FSH24, while inhibition of MAPK and PI3K enhanced FSH24-dependent follicle growth. These data support the proposition that FSH21/18 and FSH24 utilize differential mechanisms to mediate their effects. Moreover, that FSH24 inhibits follicle growth primarily via either a combination of MAPK and PI3K or the cross-talk between both pathways. Indeed, FSH has been previously shown to be able to activate PI3K and MAPK signaling in both PKA-dependent and PKA-independent manners. Additionally, cross-talk between gonadotropin hormone receptors and receptor tyrosine kinases and the downstream activation of MAPK and PI3K signaling are linked to ovulation and folliculogenesis (59, 60). When investigating apoptotic markers such as *Caspase3*, U0126 was most effective at modulating FSH24-dependent *Caspase3* expression, highlighting that FSH24-induced apoptosis may occur via MAPK signaling.

Our data suggest that the nature of FSH glycosylation modulates the follicular cellular environment to regulate follicle growth and survival. Herein, FSH ratios that mimic the profile found with aging increased follicle apoptosis and decreased steroidogenic and folliculogenic associated gene expression. They may have important implications for FSH biosimilars utilized in ovarian stimulation treatment regimens during IVF. Currently, recombinant FSH used during in vitro fertilization (IVF) protocols is primarily made up of fully glycosylated FSH, similar to FSH24. Our data suggest that a cocktail of FSH21/18:FSH24 which mimics the reproductive prime may be beneficial for follicle growth and survival. However, it must be considered that IVF is carried out on antral follicles, which have undergone hormonal priming, resulting in different responses to FSH treatment. An additional important point to address in future studies is the effect of FSH glycoforms on follicle function across ovarian life course. This current study was performed in prepubertal noncycling mice, therefore it will be necessary to determine the role of gonadotropin priming and ovarian aging. In the context of IVF, this is particularly important given the increase in female age undergoing IVF treatment and declining success rates with age (61–64). Additionally, these experiments were performed in an isolated culture, devoid of intraovarian and endocrine cross-talk, that in vivo studies would bring. Although eliminating this variable enables a more clear-cut picture of the functional role of each glycoform in the isolated follicle, understanding the endogenous regulation is an important next step. A final limitation is that repeated measures analysis could not be used with our experiment; in this experimental set up we could not track which follicle came from which mouse.

In conclusion, FSH glycoforms differentially modulate follicle growth and survival via distinct signal and gene signatures. Our findings provide potential mechanisms by which the aging-dependent ratiometric changes in FSH21/18:FSH24 direct microenvironment changes that move from promoting follicle growth and survival, to favoring atresia. These findings have potential therapeutic implications for the design of novel ovarian stimulation regimens for IVF, to improve the success rate in aging women.

## Data Availability

The original contributions presented in the study are included in the article/Supplementary Material. Further inquiries can be directed to the corresponding author.

## Abbreviations

ANOVA: analysis of variance
cAMP: cyclic adenosine monophosphate
FSH: follicle-stimulating hormone
GC: granulosa cell
IVF: in vitro fertilization
PBS: phosphate-buffered saline
PKA: protein kinase A
qRT-PCR: quantitative reverse transcription polymerase chain reaction
